# On the Differential Diagnosis of Swellings in the Neck

**Published:** 1894-12-22

**Authors:** Alexander Miles

**Affiliations:** Surgical Tutor, Royal Infirmary, Edinburgh


### Medical Progress and Hospital Clinics.

[*The Editor will be glad to receive offers of co-operation and contributions from members of the profession. All letters should be addressed to* The Editor, The Lodge, Porchester Square, London, W.]

### ON THE DIFFERENTIAL DIAGNOSIS OF SWELLINGS IN THE NECK.

By Alexander Miles, M.D., F.R.C.S., Ed., Surgical Tutor, Royal Infirmary, Edinburgh.

(*Continued from page* 188.)

### II.—Solid Swellings.

In a previous paper we have considered the chief points in the differential diagnosis of fluid swellings in the neck, and the general remarks there made with regard to the anatomical arrangement of parts, and its bearing on diagnosis, have an equal, if not greater importance, when applied to solid tumours. There are few tumours known to pathologists which may not occur in the region of the neck, but we must here confine our attention to those more frequently met with.

**Glandular Swellings.**—Undoubtedly the most common enlargements in this region are those of lymphatic glands, which are here numerous and widely distributed. (*a*) Acute Adenitis.—The marked evidence of an active inflammatory process, the situation of the inflamed structure, and the presence of some obvious cause of irritation will usually prevent an acute adenitis being mistaken for anything else. (*b*) Chronic adenitis. In investigating a case of chronic adenitis in the neck, two factors have to be borne in mind—the constitutional element, and the local determining irritant. Such swellings are more common in tubercular subjects than in others, and the evidence, direct or collateral, of this diathesis must be sought for. In addition to this predisposition, however, some source of infection in the area which the afferent lymphatics drain will usually be found. The condition of the teeth, the tongue, mouth, tonsils, pharynx, and nasal cavities must be observed The scalp or the middle ear also may be the starting-point of such glandular inflammations. A few cases remain in which no apparent cause of irritation can be made out, but the wide area of supply and the unexplorable nature of some parts of the adjacent cavities render it probable that a local irritation exists in all. Chronically enlarged cervical glands may be numerous, small, separate, and of uniform consistence, or on the other hand may be matted together into a single mass, at some points firm, at others soft and even fluctuating. Clinically their size, shape, and consistence vary so much as to be of uncertain value as points in differential diagnosis. (*c*) Syphilitic enlargement. The glands of the neck are often, but by no means always, slightly enlarged, hard, movable, and painless in constitutional syphilis. Their diagnosis is easy in the presence of a history and other evidences of the general malady. (*d*) Cancerous glands: (1) Primary cancer in a lymph gland is very rare. When it does occur it early involves the skin and fungates, at the same time as it spreads deeply among the tissues of the neck, causing much general depression and great pain, as well as symptoms of pressure on the air and food passages. (2) Secondary infection is much more common. Malignant tumours of the mouth, lips, tongue, and larynx are sooner or later followed by an enlargement of the cervical glands. The size, rapidity of growth, and pressure effects vary greatly in different cases, and the primary lesion at once points to the nature of the glandular swelling. (*e*) Hodgkin's disease is accompanied by a malignant overgrowth of lymphatic glands. This condition is known by various names—lympho-sarcoma, lymphadenoma, malignant lymphona, &c., and is a general systemic disease, whose first local manifestation is usually a painless swelling of one or more of the glands of the neck. These glands increase in size and numbers until a large mass occupies the cervical region, and at the same time similar swellings appear in the axillæ, groins, &c. The spleen enlarges and the patient suffers from a profound anæmia. It affects young adults and children most frequently, and in the early stages is exceedingly difficult to distinguish from tubercular adenitis.

**Nævi.**— Any form of nævus may occur in the neck, and the clinical features are in no way peculiar. The most difficult to diagnose are those which are subcutaneous and placed in relation to the sterno-mastoid muscle, where they are liable to be mistaken for glandular tumours.

**Fatty Tumours.**—The common encapsulated fatty tumour is readily distinguished in the neck by the same characters which mark it elsewhere—a soft, rounded, lobulated, and painless swelling, with puckering of the skin when stretched over it. The diffuse fatty tumour is occasionally met with in this region, and gives rise to much deformity.

**Tumours of Muscle.**—The swelling of muscle most frequently seen in the neck is the gumma of tertiary or inherited syphilis, which may attack any muscle, but usually appears in the sterno-mastoid. It is to be distinguished by its mode of onset, the presence of a specific history, and, if uncertainty still exist, by the effect of potassium iodide internally administered. In infants, a few days after birth, especially where the labour has been a difficult one, swellings of the sterno-mastoid are frequently observed. They are due to a rupture of the muscle, with effusion of blood into its substance, and they not infrequently produce wry-neck.

**Thyroid Tumours.**—The anatomical position, the clinical history, and the place of abode of the patient are all to be considered. The characteristic shape, the tension and consistence, and the fact that the tumour moves during deglutition, will differentiate enlargements of the thyroid body from other cervical swellings.

**Other Swellings.**—Traumatic swelllings and such conditions as boils and carbuncles, give rise to no difficulties in diagnosis; and the rare forms of growth, such as fibroma, enchondroma, and osteoma, need not be more than mentioned.

